# Supplementary material for: Novel Perbutyrylated Glucose Derivatives of (–)-Epigallocatechin-3-Gallate Inhibit Cancer Cells Proliferation by Decreasing Phosphorylation of the EGFR: Synthesis, Cytotoxicity, and Molecular Docking
Source: Molecules. 2021 Jul 19;26(14):4361. doi: 10.3390/molecules26144361 (PMC8306927; doi:10.3390/molecules26144361)

Supporting Information

Novel per-butyrylated glucoses derivatives of (-)-epigallocatechin-3-gallate inhibit cancer cells proliferation by decreasing phosphorylation of the EGFR: Synthesis, cytotoxicity and molecular docking

Ya Wang [1,2,a], Xiao-Jing Shen [3,a], Fa-Wu Su [4,a], Yin-Rong Xie [1,2], Li-Xia Wang [1,2], Ning Zhang [1,2], Yi-Long Wu [1,2], Yun Niu [1,2], Dong-Ying Zhang [1], Cheng-Ting Zi [1,*], Xuan-Jun Wang [1,*], Jun Sheng [1,2,*]

[1] Key Laboratory of Pu-er Tea Science, Ministry of Education, College of Science, Yunnan Agricultural University, Kunming, 650201, China; wangya9188@126.com; xyr351527080@163.com; wanglixia0315@163.com; zn1193594574@163.com; ynau_wuyl@126.com; Niuyun0429@163.com; 757664982@qq.com; zichengting@126.com; xuanjunwang@qq.com; shengj@ynau.edu.cn

[2] College of Food Science and Technology, Yunnan Agricultural University, Kunming, 650201, China; wangya9188@126.com; xyr351527080@163.com; wanglixia0315@163.com; zn1193594574@163.com; ynau_wuyl@126.com; Niuyun0429@163.com; shengj@ynau.edu.cn

[3] Party Committee of Organ, Yunnan Agricultural University, Kunming, 650201, China; sxj243@sina.com

[4] State Key Laboratory for Conservation and Utilization of Bio-Resources in Yunnan, Yunnan Agricultural University, Kunming, 650201, China; su_faw@126.com

[a] These authors contributed equally to this work

Table S1. The characteristic ^{1}H-NMR and ^{13}C-NMR of compounds **7**–**12**

Table S2. The energies of the binding between EGFR and compounds **4**, **11** and **12**

Figure S1. The details of the binding between EGFR and EGCG (**4**)

Figure S2. ^{1}H-NMR spectra of compound **6**

Figure S3. ^{13}C-NMR spectra of compound **6**

Figure S4. ^{1}H-NMR spectra of compound **7**

Figure S5. ^{13}C-NMR spectra of compound **7**

Figure S6. HMBC spectra of compound **7**

Figure S7. ^{1}H-NMR spectra of compound **8**

Figure S8. ^{13}C-NMR spectra of compound **8**

Figure S9. HMBC spectra of compound **8**

Figure S10. ^{1}H-NMR spectra of compound **9**

Figure S11. ^{13}C-NMR spectra of compound **9**

Figure S12. ^{1}H-NMR spectra of compound **10**

Figure S13. ^{13}C-NMR spectra of compound **10**

Figure S14. ^{1}H-NMR spectra of compound **11**

Figure S15. ^{13}C-NMR spectra of compound **11**

Figure S16. ^{1}H-NMR spectra of compound **12**

Figure S17. ^{13}C-NMR spectra of compound **12**

Table S1. The characteristic ^{1}H-NMR and ^{13}C-NMR of compounds **7**–**12**.

**Compounds**	**H-1‴**		**C-1‴** δ (ppm)	**H-1''''**		**C-1''''** δ (ppm)
	δ (ppm)	$J_{1'''\text{-}2'''}$(Hz)		δ (ppm)	$J_{1''''\text{-}2''''}$(Hz)	
**7**	—	—	—	4.9	9.0	102.7
**8**	4.7	9.5	107.0	4.5	9.5	107.3
**9**	—	—	—	4.7	9.0	108.2
**10**	4.7	9.5	107.0	4.5	9.5	107.8
**11**	—	—	—	4.9	9.0	102.7
**12**	4.7	9.5	107.1	4.6	9.5	107.8

Table S2. The energies of the binding between EGFR and compounds **4**, **11** and **12**.

**Compd.**	**(vdW+Hbond+desolv)energy(kcal/mol)**	**Electrostatic energy (kcal/mol)**	**Total internal energy (kcal/mol)**	**The best docking energy (kcal/mol)**	**Inhibition constant (nM)**
**4**	–7.7	–1.1	–3.8	–5.0	159.2
**11**	–8.1	–0.4	–10.9	2.4	1754.3
**12**	–8.4	–0.09	–10.4	1.91	455.5

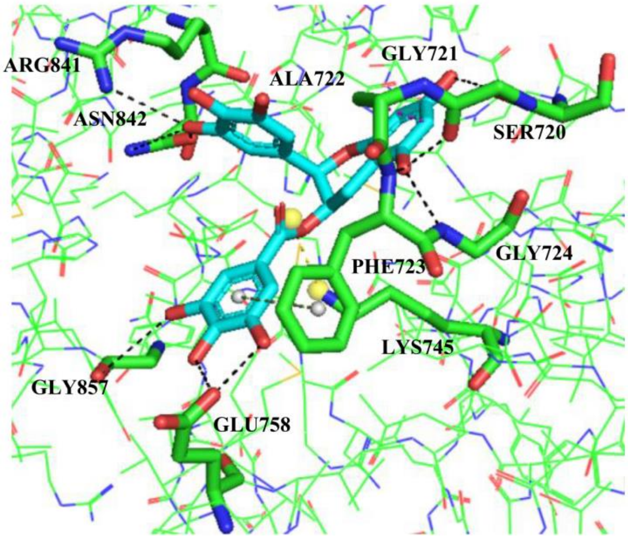

Figure S1. The details of the binding between EGFR and EGCG (**4**): the protein is shown as lines; ligand and the key residues are shown as sticks (ligand color: C cyan, N blue, O red and polar hydrogen); hydrogen bonds are shown as black dotted lines; hydrophobic interactions are shown as red dotted lines; π-stacking is shown as gray dotted lines; salt bridges were shown as yellow dotted lines.

Figure S2. ^{1}H-NMR spectra of compound **6**

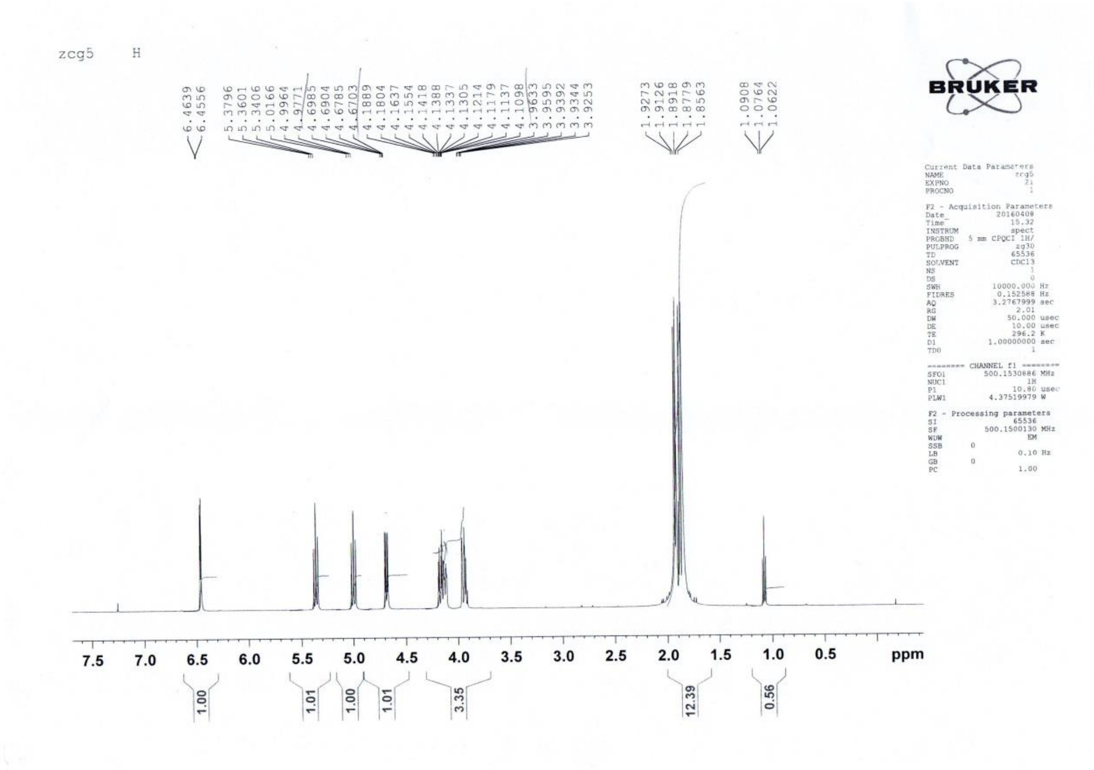

Figure S3. ^{13}C-NMR spectra of compound **6**

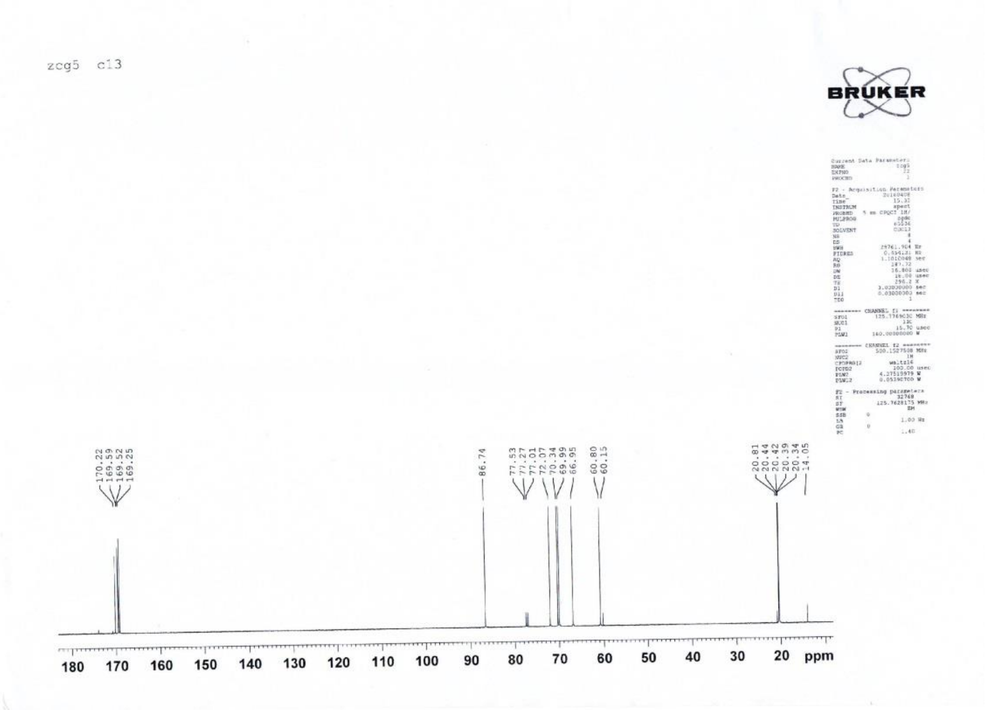

Figure S4. ^{1}H-NMR spectra of compound **7**

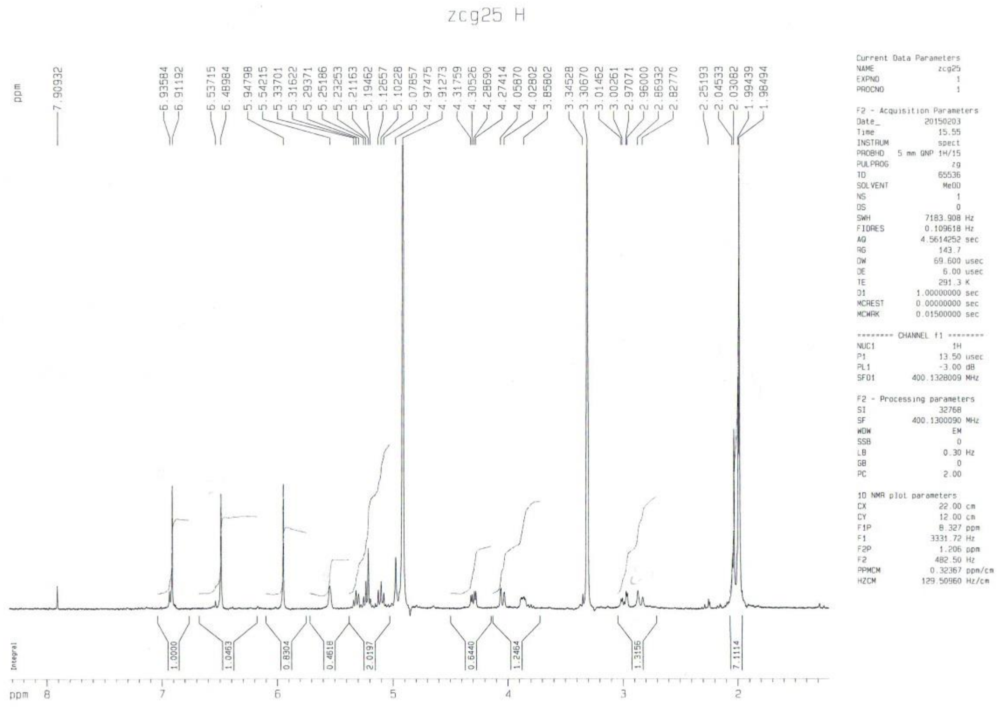

Figure S5. ^{13}C-NMR spectra of compound **7**

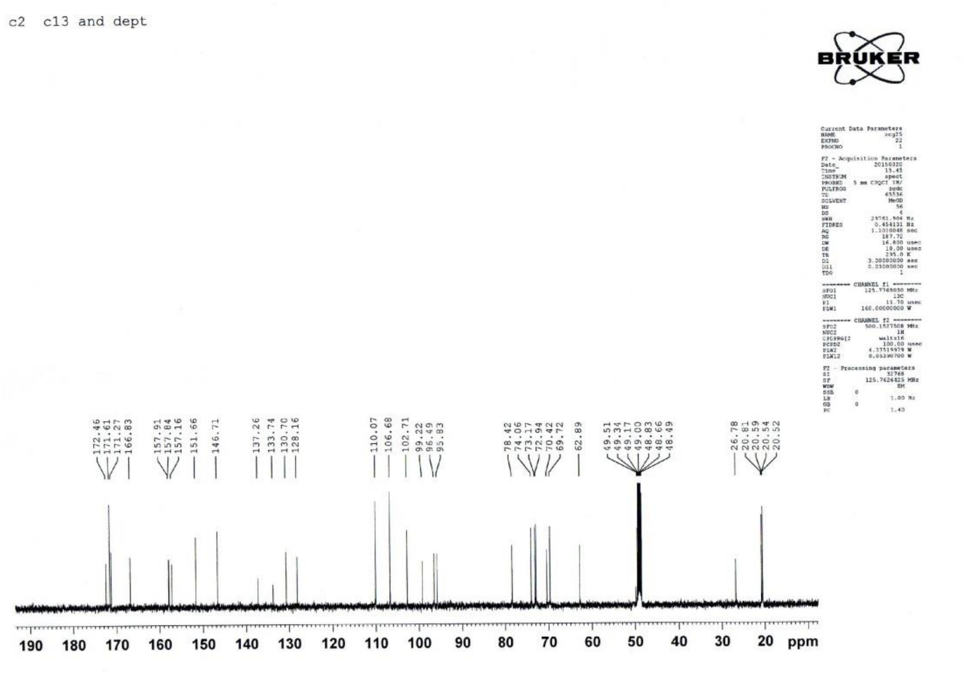

Figure S6. HMBC spectra of compound **7**

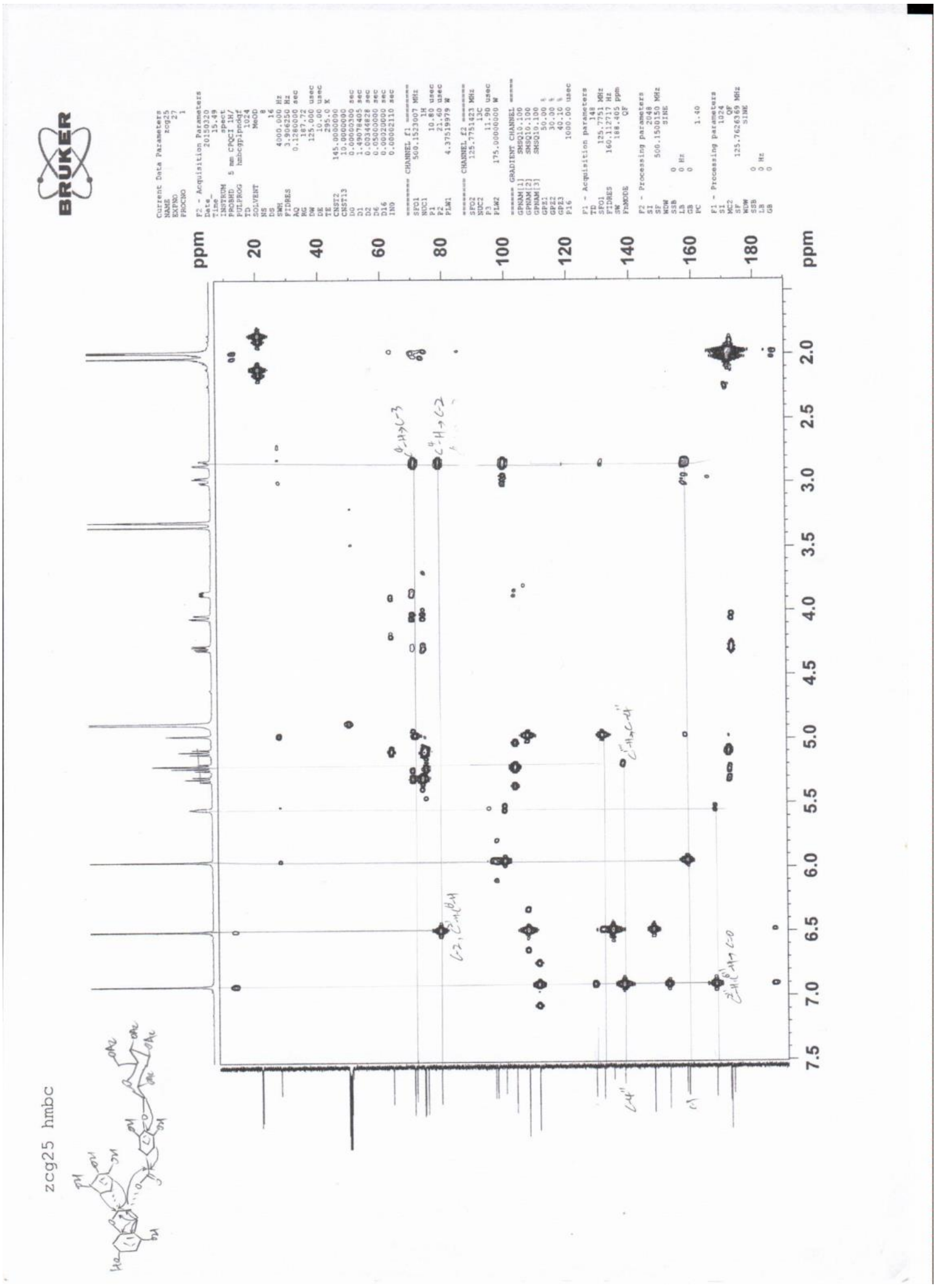

Figure S7. ^{1}H-NMR spectra of compound **8**

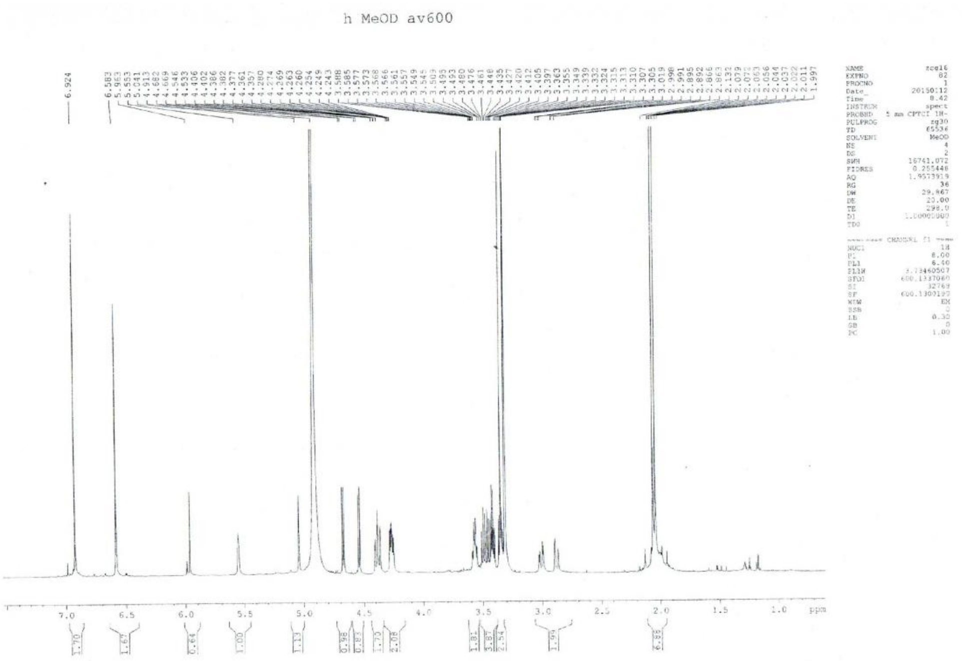

Figure S8. ^{13}C-NMR spectra of compound **8**

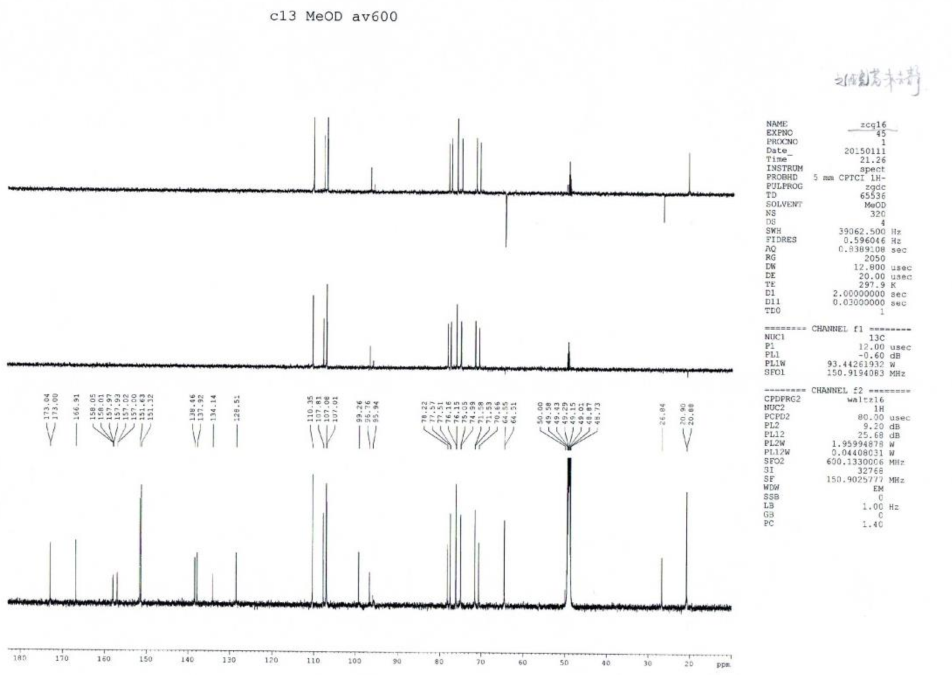

Figure S9. HMBC spectra of compound **8**

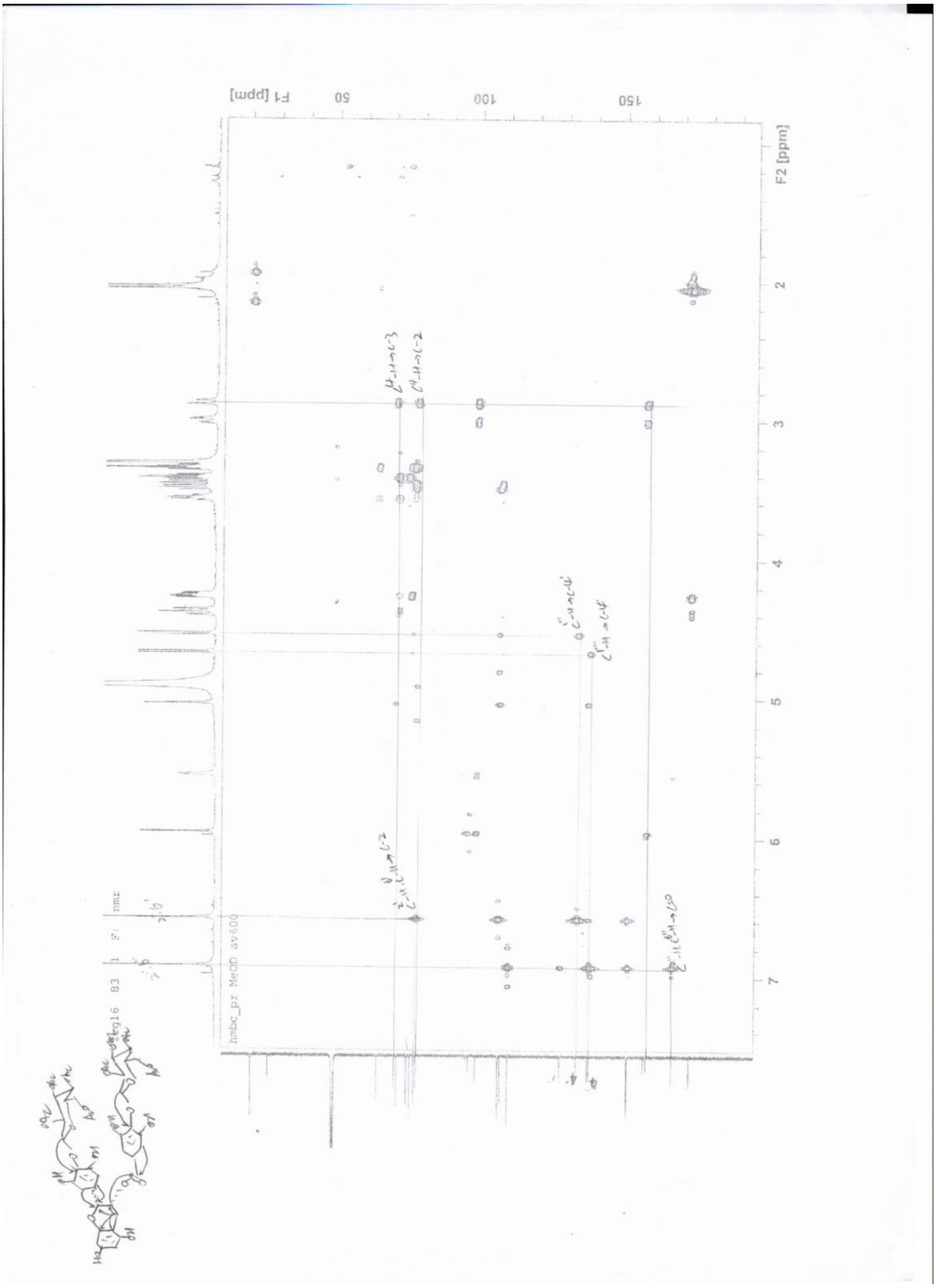

Figure S10. ^{1}H-NMR spectra of compound **9**

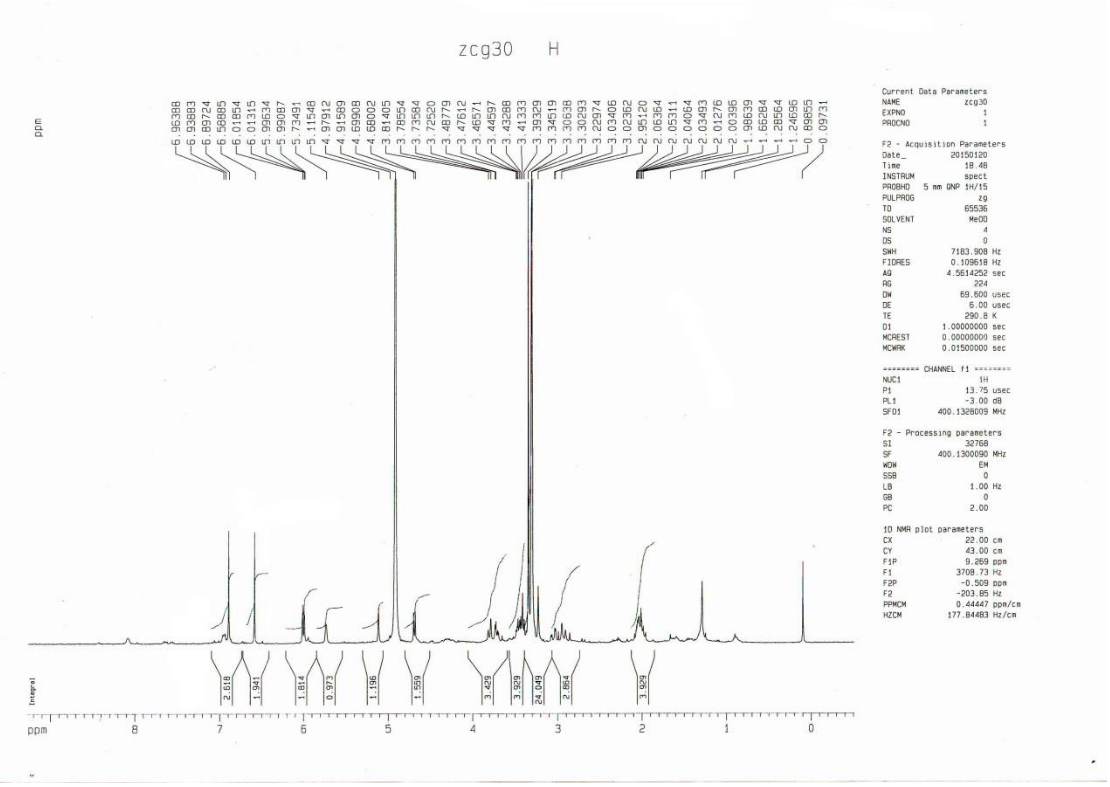

Figure S11. ^{13}C-NMR spectra of compound **9**

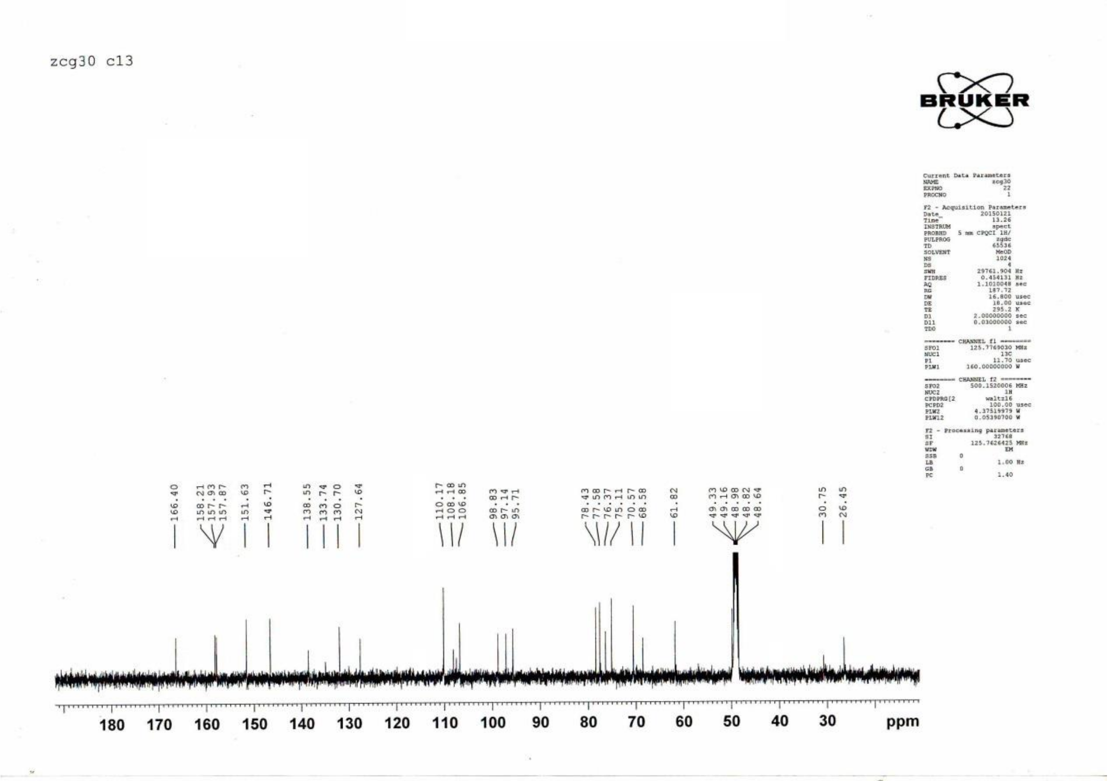

Figure S12. ^{1}H-NMR spectra of compound **10**

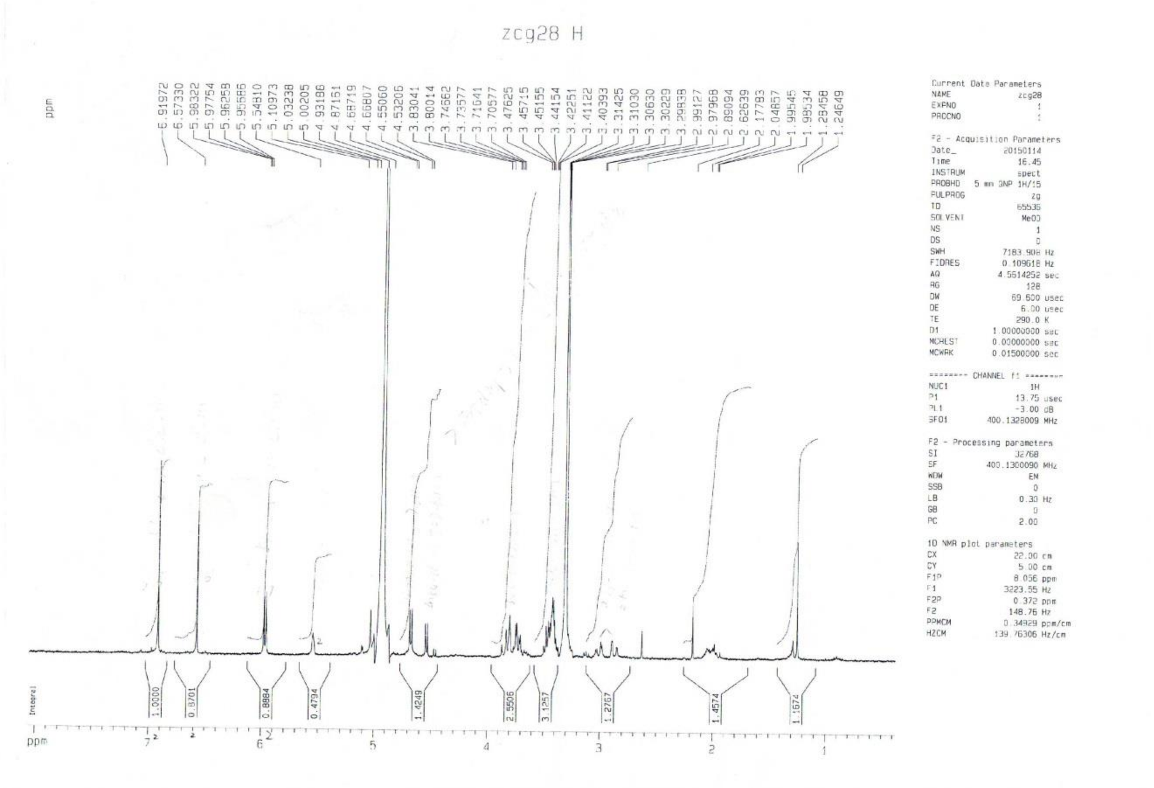

Figure S13. ^{13}C-NMR spectra of compound **10**

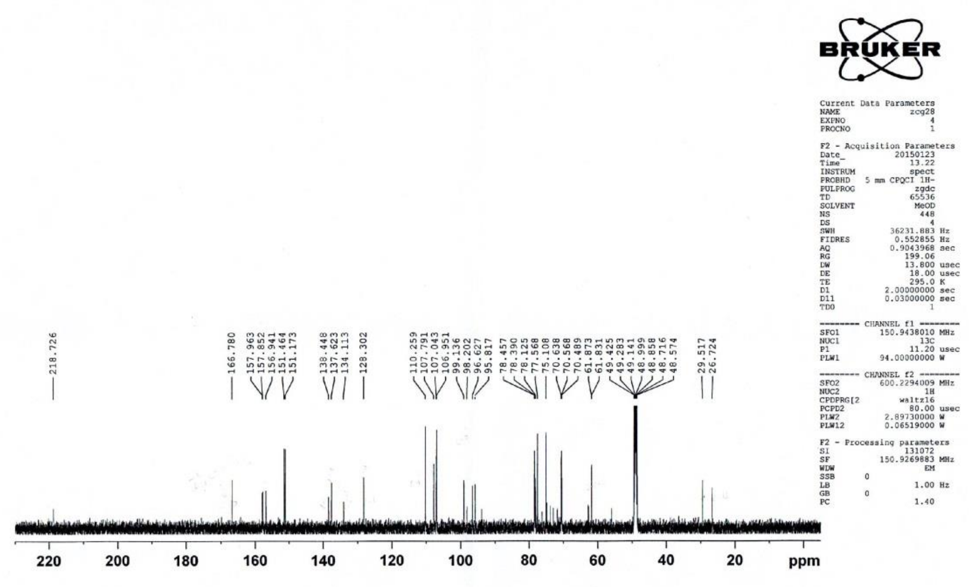

Figure S14. ^{1}H-NMR spectra of compound **11**

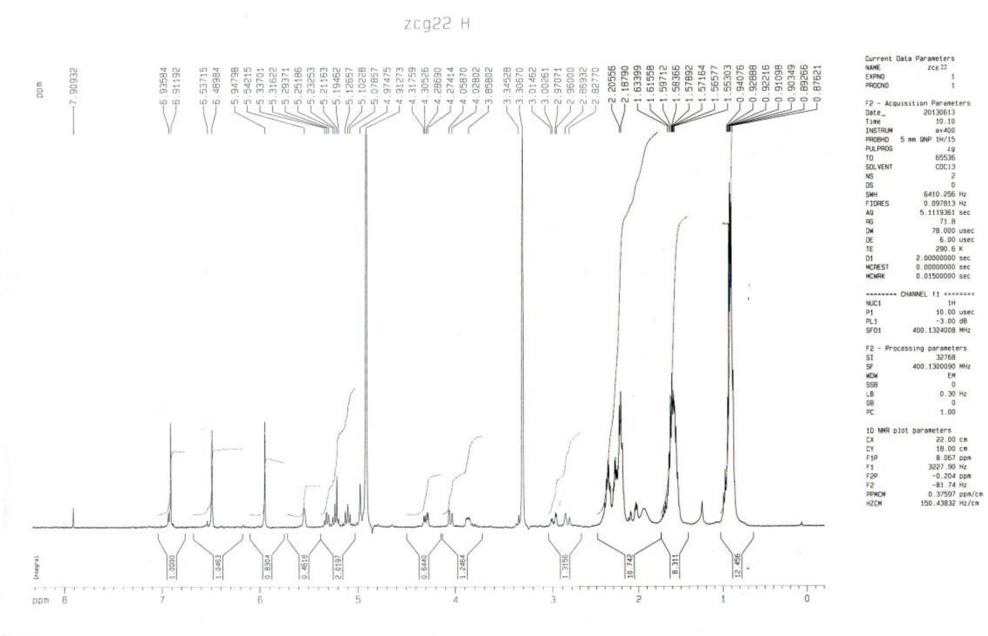

Figure S15. ^{13}C-NMR spectra of compound **11**

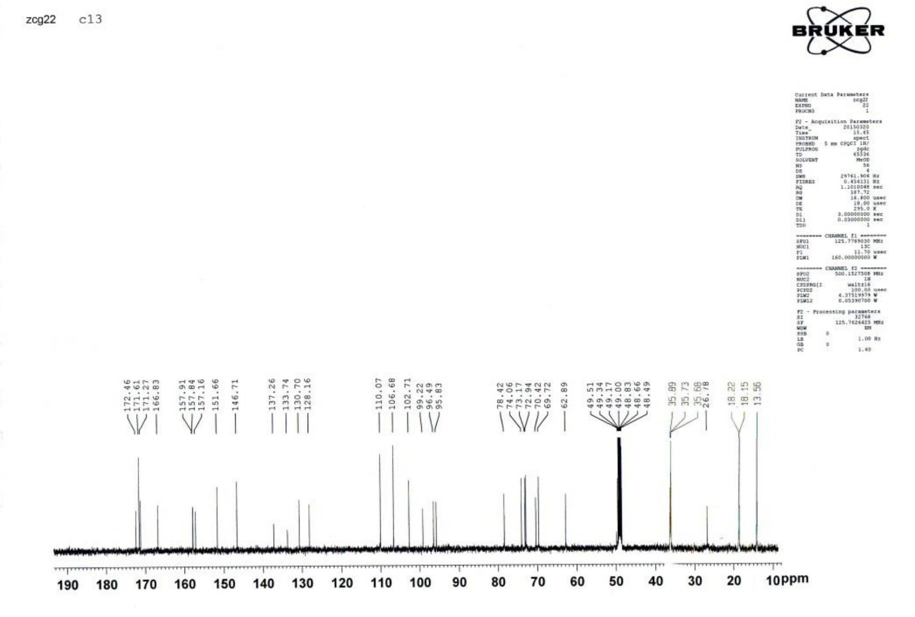

Figure S16. ^{1}H-NMR spectra of compound **12**

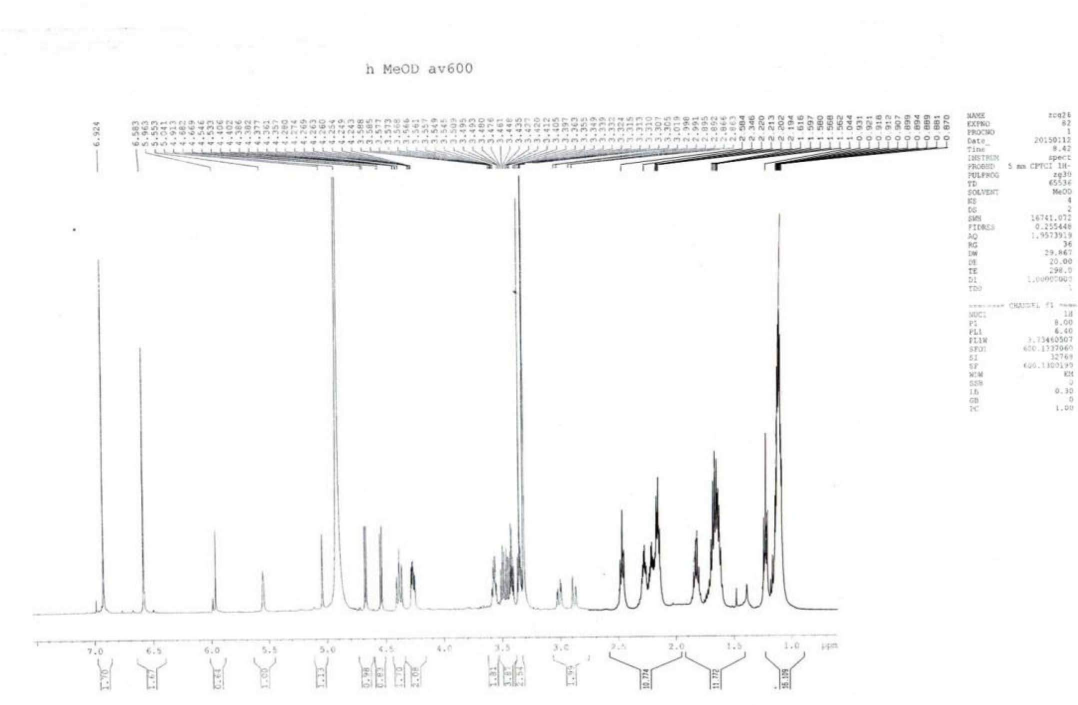

Figure S17. ^{13}C-NMR spectra of compound **12**

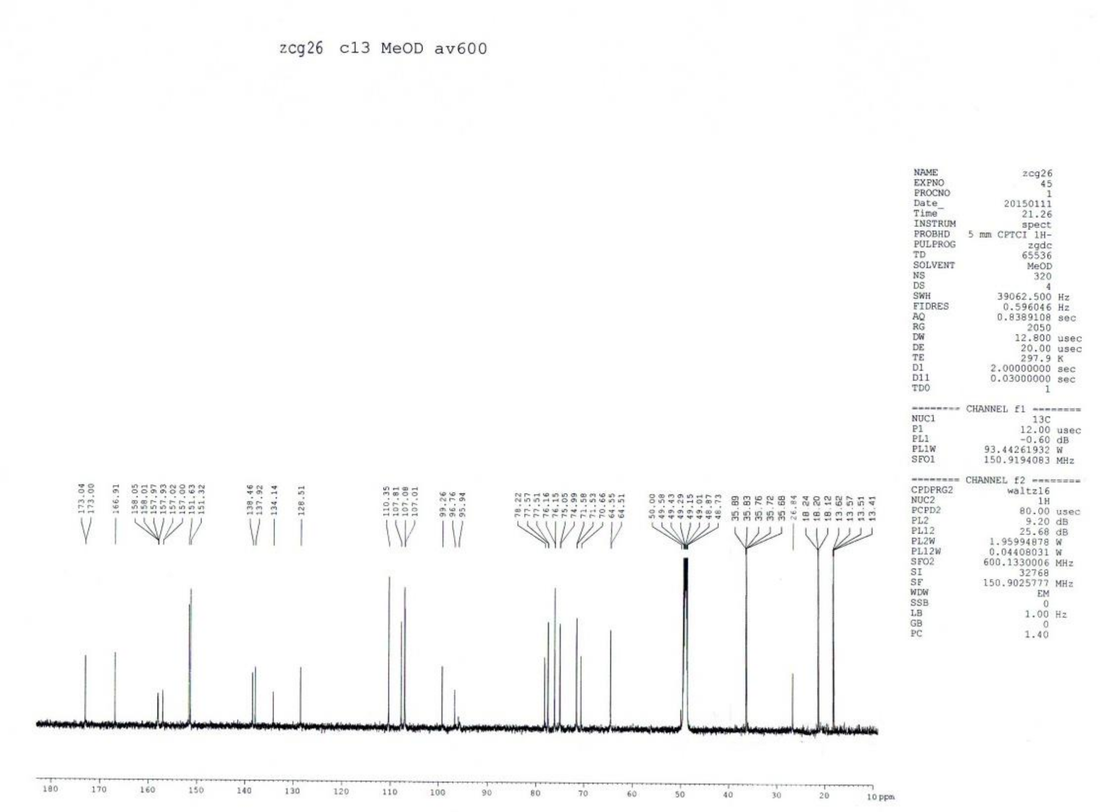

Supplement: Supplementary file 1 [file molecules-26-04361-s001.zip › molecules-1229590-supplementary.pdf]
